# The Unusual Clinical Presentation of Eosinophilic Gastroenteritis and Its Response to Mepolizumab: Two Case Reports

**DOI:** 10.7759/cureus.72589

**Published:** 2024-10-28

**Authors:** Mahmoud A Kiblawi, Talha Malik, Mazin M Edan

**Affiliations:** 1 Internal Medicine, Sheikh Shakhbout Medical City, Abu Dhabi, ARE; 2 Gastroenterology and Hepatology, Mayo Clinic Arizona, Phoenix, USA; 3 Gastroenterology and Hepatology, Sheikh Shakhbout Medical City, Abu Dhabi, ARE

**Keywords:** biologic agents, eosinophilic gastrointestinal disorder, eosinophilic infiltrates, gastro-intestinal, il-5

## Abstract

Eosinophilic gastroenteritis (EGE) is a rare digestive disorder that affects individuals of all ages, characterized by eosinophilic infiltration in the stomach and intestines. Various risk factors contribute to EGE, and its pathogenesis remains poorly understood. The definitive diagnosis relies on histological examination of the involved gastrointestinal (GI) tract, revealing evidence of eosinophilic infiltration. We present two case reports of young males diagnosed with EGE who achieved good outcomes with early management, including steroids and mepolizumab. The use of a biologic agent, anti-IL5, helped reduce the use of steroids and both patients were in complete remission based on GI symptom assessments. The clinical presentation of EGE is non-specific, which can make diagnosis challenging. Currently, there is no definitive consensus on the optimal treatment for EGE, and management remains primarily empirical. Limited data are available on EGE, and these cases may help provide additional insights into the diagnosis and management of this rare disease.

## Introduction

Eosinophilic gastroenteritis (EGE) is a rare inflammatory disorder characterized by the infiltration of eosinophils in the gastrointestinal (GI) tract [1,2]. Limited data exist on its prevalence in the Middle East, including the UAE. Studies conducted in the United States of America (USA) estimate the overall prevalence of EGE to be 5.1-8.4 per 100,000 persons [3]. EGE can manifest at any age but is more prevalent in the younger populations, with a higher predisposition in females [3]. It has a peak onset typically occurring between the third and fifth decades of life [4]. Various risk factors contribute to EGE, including higher socioeconomic status, Caucasian race, obesity, autoimmune diseases, genetics, and allergic disorders [1,2]. Several case reports and studies in the literature have explored the nature of the disease, its course, and its outcomes. Currently, there is no definitive consensus on the optimal treatment for EGE, and management remains primarily empirical [4]. We present two case reports of young males diagnosed with EGE. The cases highlight the various clinical presentations of EGE, with a particular focus on management using mepolizumab.

This article was previously presented at the Second International UAE Rare Disease Society Congress in February 2024 and the MENA Congress for Rare Diseases in May 2024, both conferences were held in Abu Dhabi, United Arab Emirates (UAE).

## Case presentation

Case 1

A 19-year-old male patient with a past medical history of bronchial asthma and atopic dermatitis was diagnosed with EGE in 2011 at the age of six. The patient's initial presentation was persistent nausea, intermittent vomiting, non-specific generalized abdominal pain, and constipation. The patient required hospitalization and underwent several laboratory investigations. His initial workup revealed elevated white blood count (WBC) of 20*10^9^, hyperesinophilia of 58%, mild C-reactive protein (CRP) elevation, normal erythrocyte sedimentation, and immunoglobulin E (Ig-E) level was elevated at 300 IU/mL (Table 1). He had a viral screening, celiac disease, and autoimmune workup which were negative (Table 2). The patient underwent bone marrow biopsy and cytogenetics which were negative for malignancy. The bone marrow revealed trilineage hematopoiesis, normal maturation of hematopoietic precursors, and significantly increased eosinophils associated with severe peripheral blood eosinophilia. He had esophagogastroduodenoscopy (EGD), and several biopsies showed significant eosinophils in the esophagus, stomach, and duodenum. The patient was diagnosed as a case of EGE based on the clinical presentation and histopathology results. He was treated symptomatically including dietary modification and required a course of steroids.

**Table 1:** Laboratory investigations on initial presentation

Laboratory investigations				
	Patient 1		Patient 2	
Test	Result	Normal range	Result	Normal range
White blood count	20 *10^9^/L	6-15 *10^9^/L	14.41 *10^^9^/L	4-11 *10^^9^/L
Hemoglobin	116 g/L	103-149 g/L	156 g/L	130-170 g/L
Platelet	344 *10^9^/L	(210-650 *10^9^/L)	252 *10^9^/L	140-400 *10^9^/L
Neutrophils	25.2%	-	35.8%	-
Lymphocytes	10%	-	9.1%	-
Monocytes	6.0%	-	4.0%	-
Eosinophils	58%	-	50.7%	-
Basophils	0.8%	-	0.4%	-
Creatinine	33 micromol/L	44-88 micromol/L	65 micromol/L	62-106 micromol/L
Aspartate aminotransferase	23 IU/L	<40 IU/L	43 IU/L	<50 IU/L
Alanine transaminase	10 IU/L	<40 IU/L	50 IU/L	<50 IU/L
Albumin	47 g/L	35-50 g/L	37 g/L	35-52 g/L
Total protein	74 g/L	66-87 g/L	66 g/L	66-87 g/L
Lactate dehydrogenase	273 IU/L	135 -225 IU/L	205 IU/L	135 -225 IU/L
Calprotectin	203 ng/mL	<50 ng/mL	1,854 mcg/g	<80 mcg/g
Lipase	-	-	107 IU/L	13-60 IU/L
BCR/ABL gene	-	-	Not detected	-
Celiac antibody	Negative	-	Negative	-
Hepatitis screening	-	-	Negative	-
Erythrocyte sedimentation rate	4 mm/hr	1-13 mm/hr	2 mm/hr	1-13 mm/hr
Immunoglobulin E (Ig E)	300 IU/mL	<100 IU/mL	269 IU/mL	<100 IU/mL
Human immunodeficiency virus (HIV) screening	-	-	Negative	-
QuantiFERON tuberculosis	-	-	Negative	-
Stool analysis, parasitic, molecular and culture	Negative	-	Negative	-
Helicobacter pylori urea breath test	-	-	Positive	-

**Table 2:** Autoimmune workup

	Patient 1		Patient 2	
Test	Result	Normal range	Result	Normal range
Antinuclear antibodies (ANA)	Negative	-	Positive	-
ANA indirect immunofluorescent (IIF)	-	-	Negative	-
Antineutrophil cytoplasmic antibodies, Cytoplasmic (C-ANCA ) proteinase 3	2 RU/mL	<=20 RU/mL	3 RU/mL	<=20 RU/mL
Perinuclear anti-neutrophil cytoplasmic antibodies (P-ANCA) Myeloperoxidase	<2 RU/mL	<=20 RU/mL	2 RU/mL	<=20 RU/mL
Double stranded DNA antibody	-	-	26.4 IU/mL	<=26.9 IU/mL
Extractable nuclear antigen (ENA) screen	-	-	>429.4 CU	<=20 CU
Smith and ribonucleoprotein (SMRNP) antibody	-	-	Negative	-
Anti-Smith (Sm) antibody	-	-	Negative	-
Anti-Sjogren's syndrome type A (SS-A) antibody	-	-	Negative	-
Anti-Sjogren's syndrome type B (SS-B) antibody	-	-	Negative	-
Scleroderma (Scl) 70 antibody	-	-	Negative	-
JO-1 antibody	-	-	Negative	-
Ro-52 antibody	-	-	Negative	-
Polymyositis-Scleroderma overlap (PM-Scl) antibody	-	-	Negative	-
Centromere protein B (CENP-B) antibody	-	-	Negative	-
Proliferating cell nuclear antigen (PCNA) antibody	-	-	Negative	-
Histones antibody	-	-	Negative	-
Nucleosomes antibody	-	-	Negative	-
Anti ribosomal protein (Rib. protein) antibody	-	-	Negative	-
Anti-mitochondrial M2 (AMA-M2) antibody	-	-	Negative	-
Dense fine speckled 70 (DFS70) antibody	-	-	Negative	-

**Figure 1:**
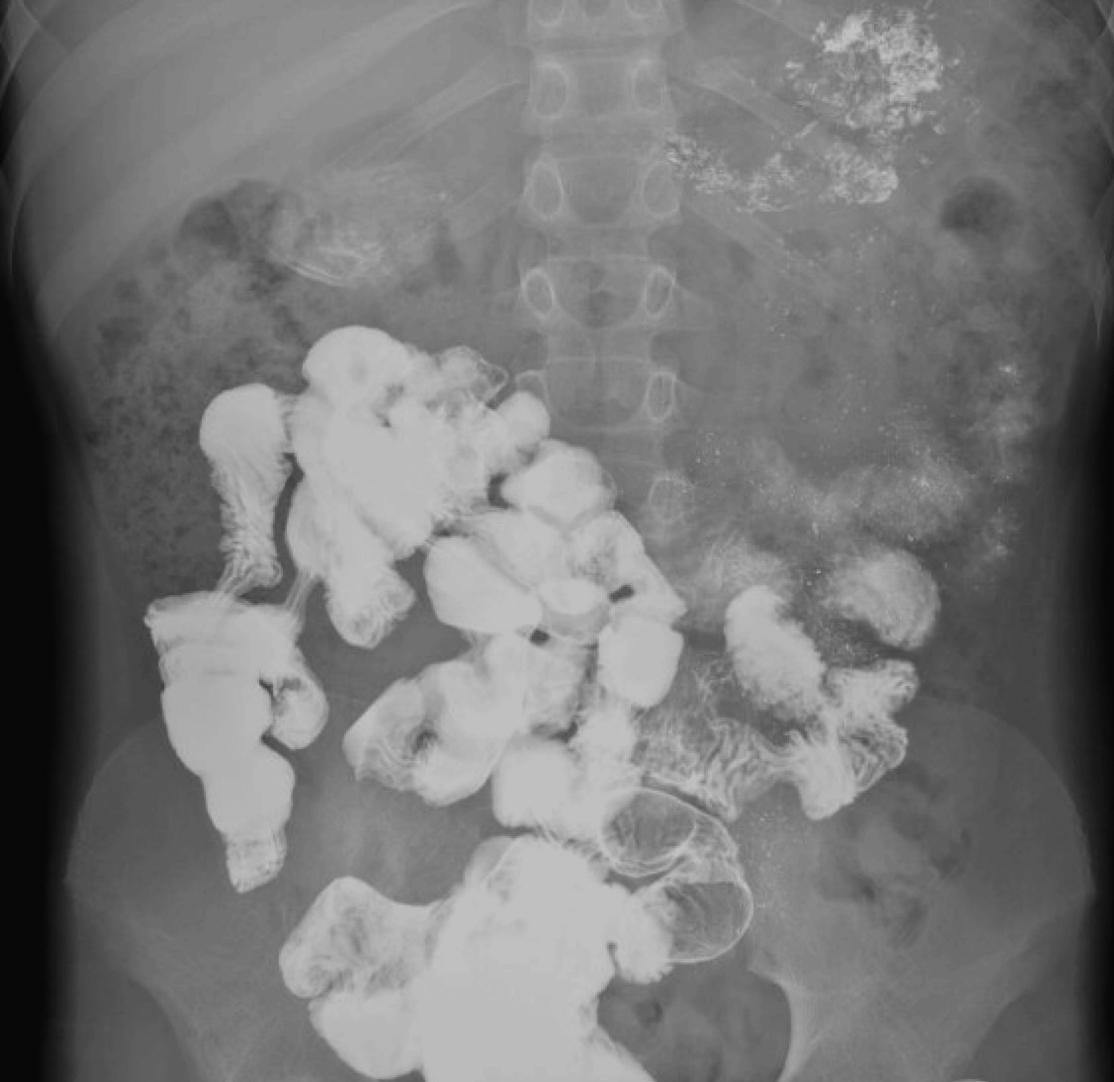
Upper GI small bowel gastrografin study post duodenal obstruction resolution

**Figure 2:**
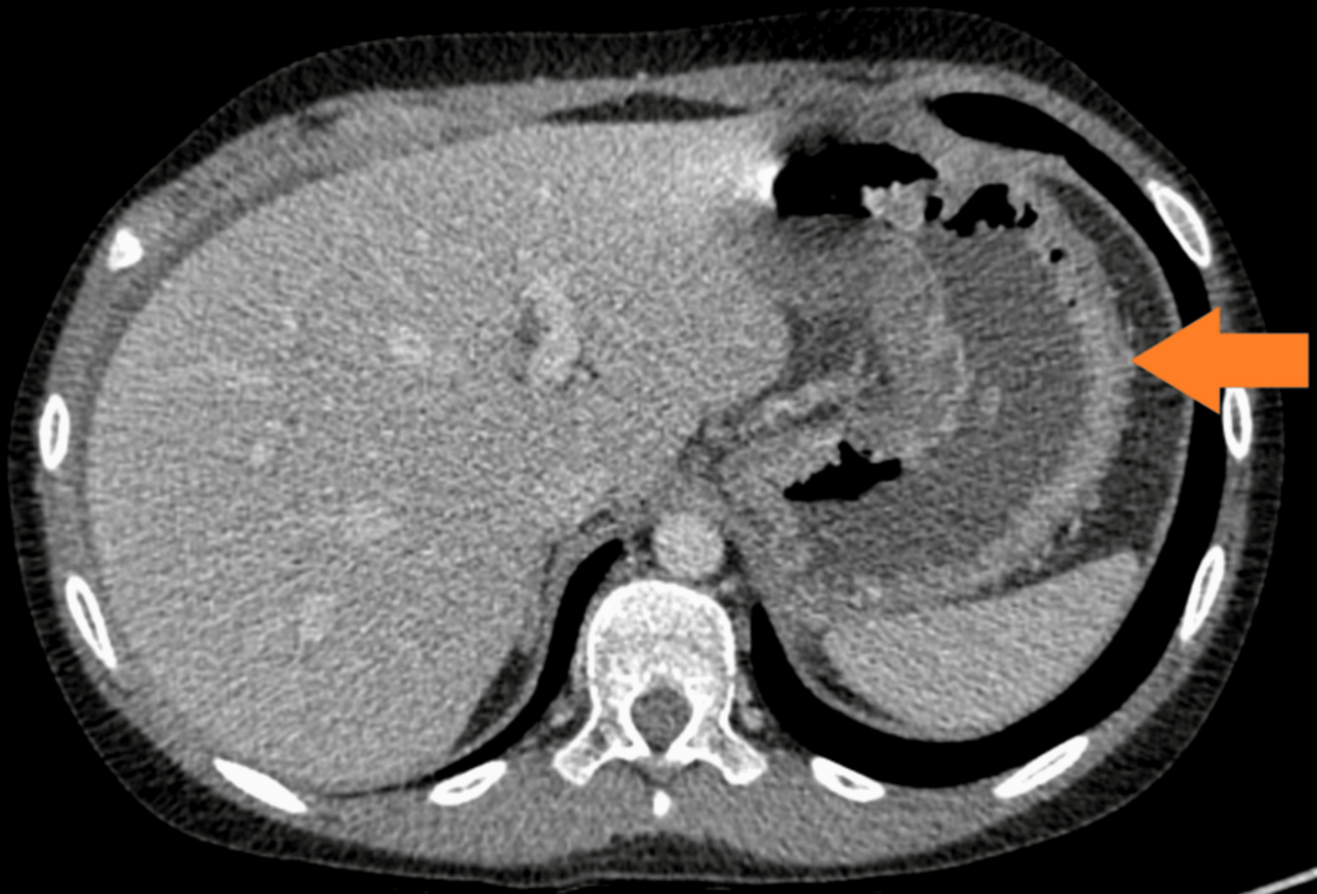
Mural thickening of the stomach wall

**Figure 3:**
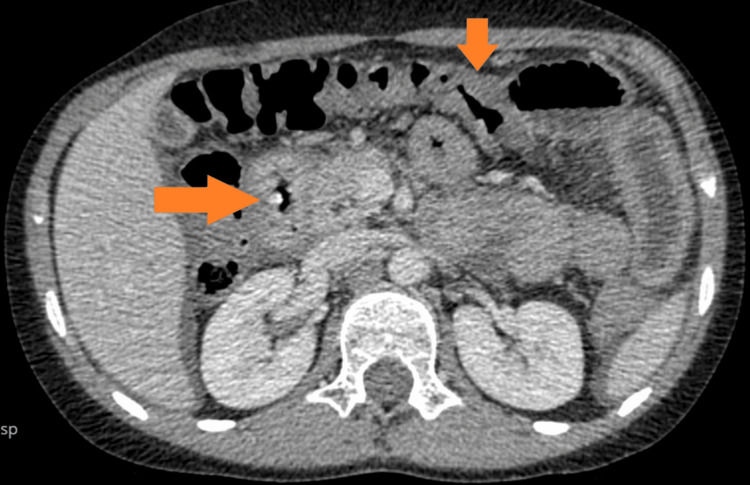
Mural thickening extending to the pyloroduodenal junction and all parts of the duodenum

**Figure 4:**
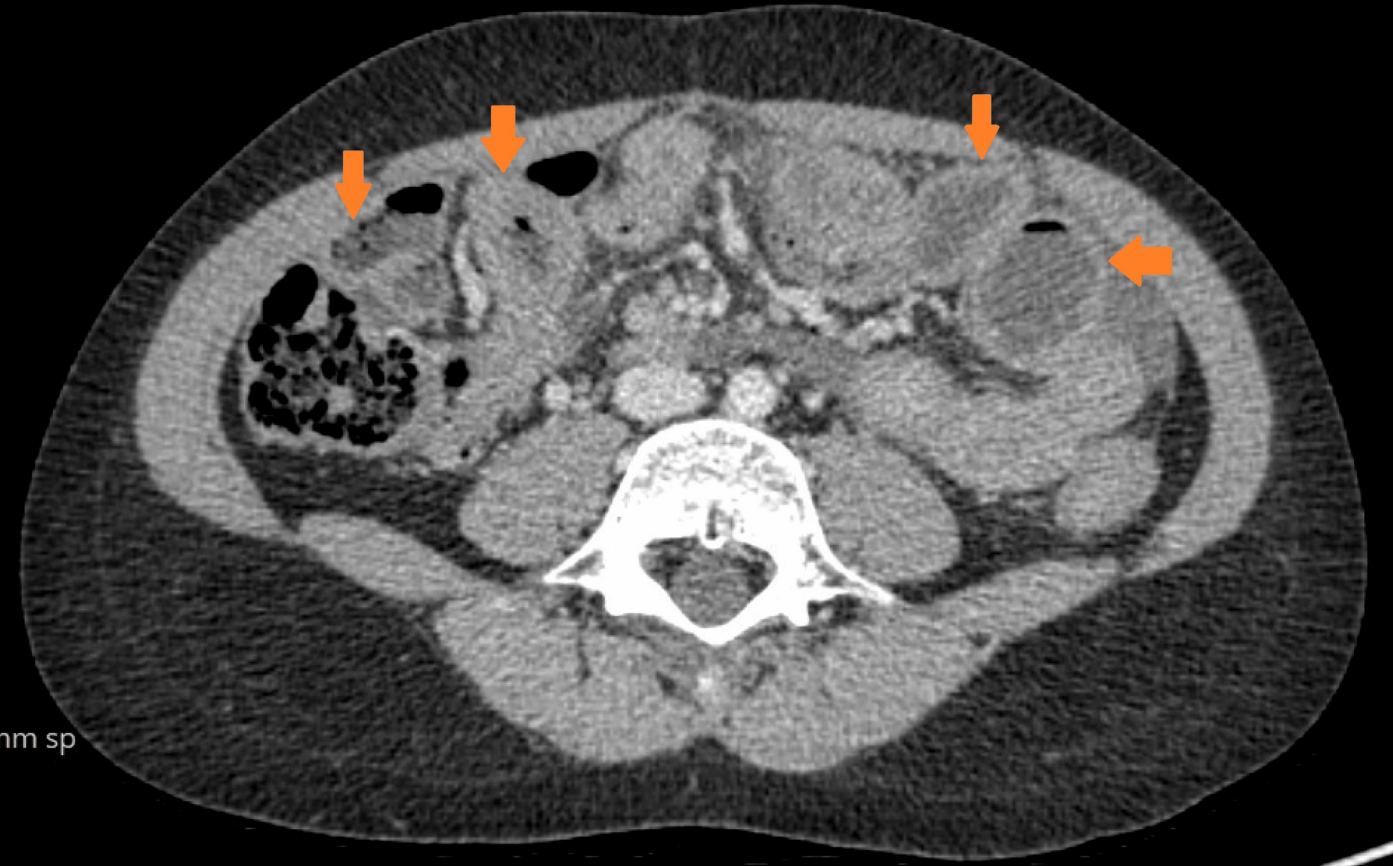
Inflammatory changes extending throughout all bowel loops

The patient kept developing recurrent exacerbations which mainly responded well to steroids. He did have EGD during the exacerbations that showed gastritis and eosinophilia. At the age of 11, he had severe symptoms of vomiting and abdominal pain. EGD showed narrowing at the level of the duodenum with the inability to pass the scope through the narrow lumen. The biopsy revealed chronic gastritis, and eosinophilic infiltration of the esophagus, and was negative for malignancy. An upper GI small bowel gastrografin (SBG) study was suggestive of duodenal obstruction. A computerized tomography (CT) of the abdomen and pelvis showed a grossly thickened duodenum obstructing the ampulla of Vater. The patient had an exploratory laparotomy which showed thickening of the duodenum and jejunum with biopsies that were inconclusive. He did respond to steroids but not to azathioprine itself. The patient had a long course of tapering steroids until the resolution of his symptoms. Several SBG studies were done a few months later and showed a resolution of the obstruction (Figure 1). However, he kept having recurrent episodes of exacerbation and at the age of 13, he had a severe flare-up of his disease requiring tapering steroid therapy to control his symptoms. A CT of the abdomen showed diffuse inflammatory changes throughout the GI tract, but no perforation or obstruction (Figures 2-4). At the age of 16, the patient was initiated on a mepolizumab dose of 100 mg SC every four weeks. The patient showed significant improvement following the first two doses of mepolizumab. He was continued on biologic agent with a regular follow-up in the GI clinic. He did not have any further disease flare or relapse. He had EGD and colonoscopy in June 2023 which were within normal limits. Histopathology from several biopsies showed resolution of the underlying inflammation and eosinophilic infiltration. His last visit was in August 2024, the patient is in clinical remission based on an assessment of his GI symptoms. The patient continues to take mepolizumab with a good response. Moreover, his underlying bronchial asthma has been better controlled without any flares.

Case 2

**Figure 5:**
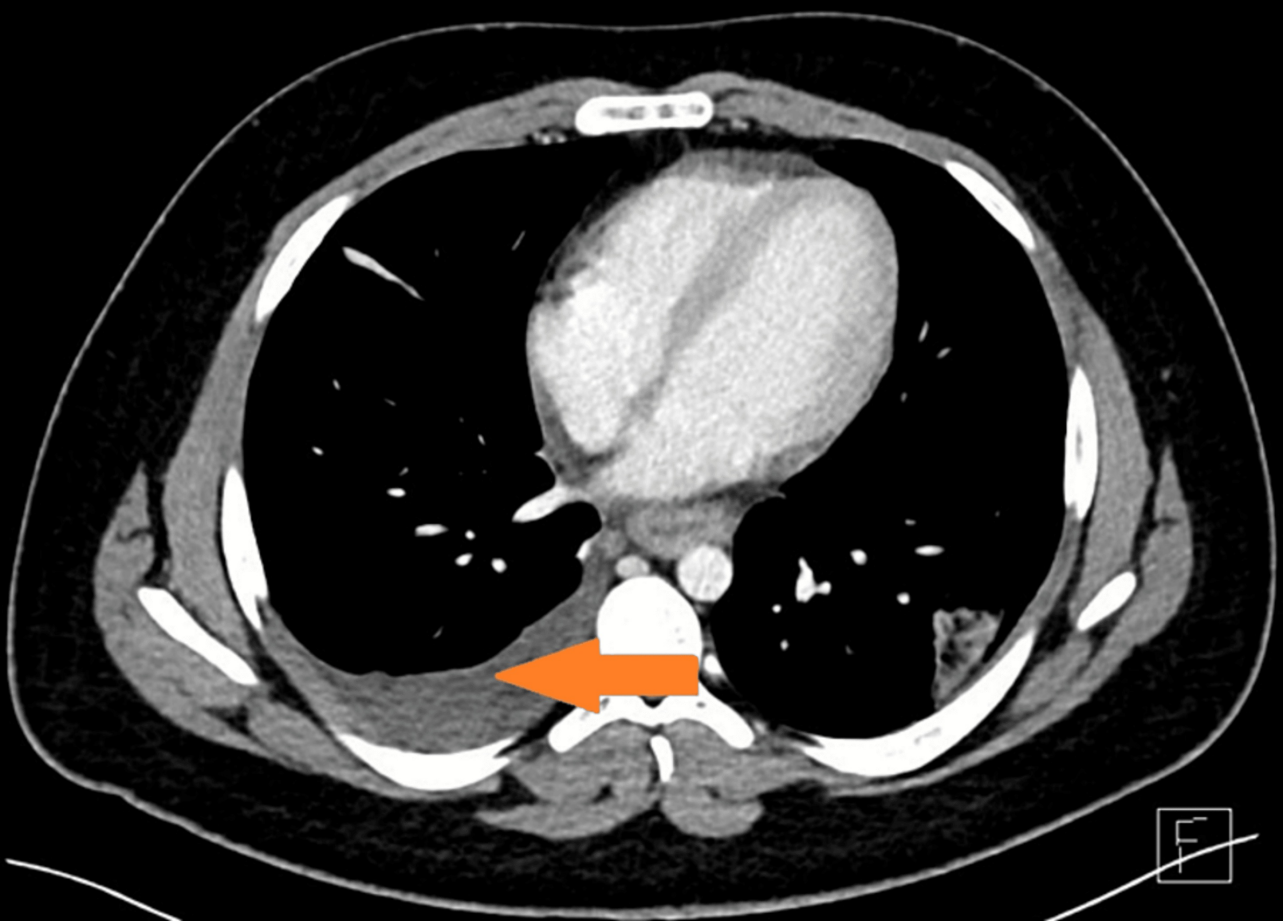
Computerized tomography of the abdomen showing pleural effusion

**Figure 6:**
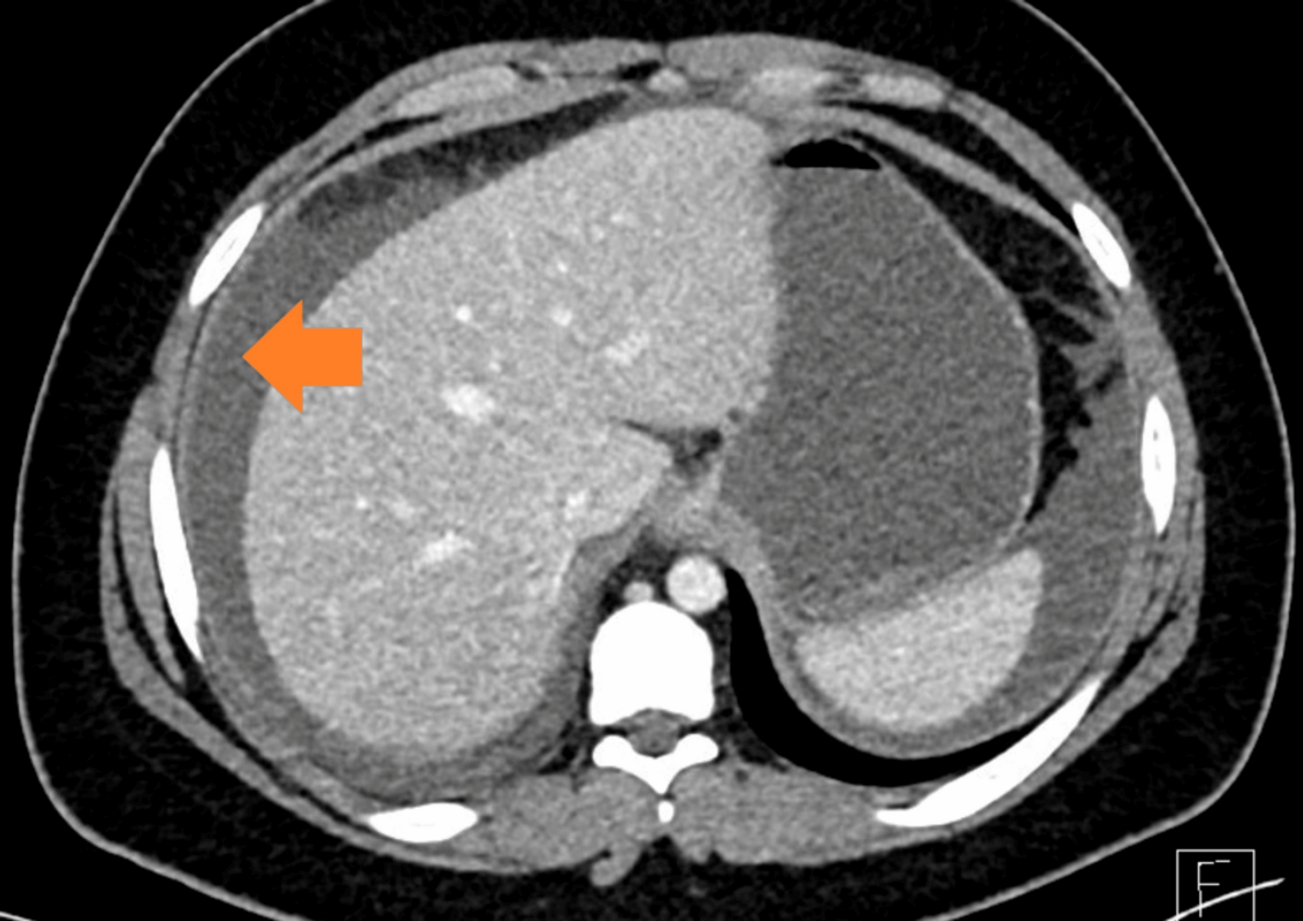
Computerized tomography of the abdomen showing ascites

**Figure 7:**
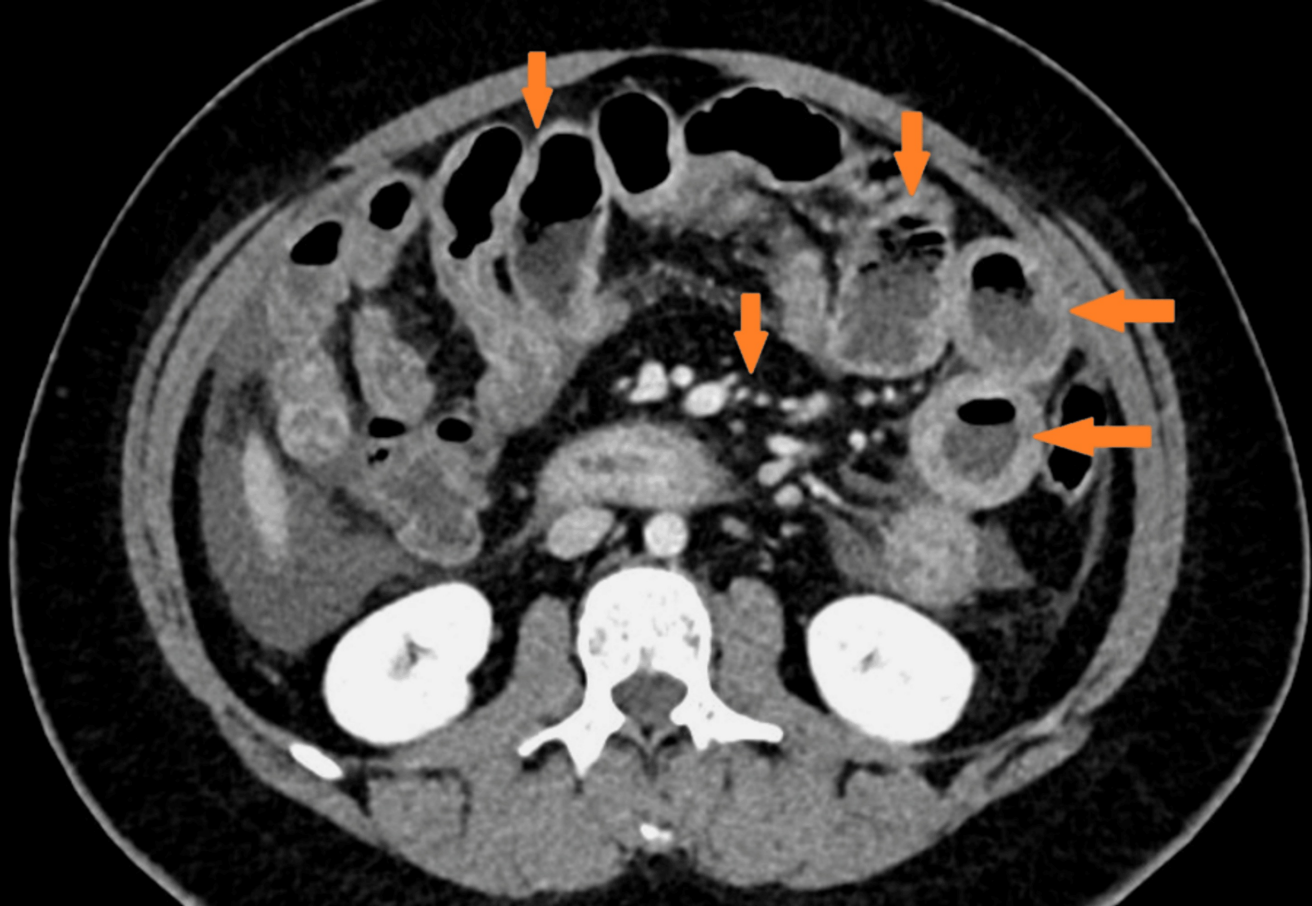
Computerized tomography of the abdomen showing bowel thickening, edema and reactive lymph nodes

An 18-year-old male patient, previously healthy, presented to the hospital with chief complaints of localized intermittent epigastric pain for one-month duration. It was associated with nausea, loss of appetite, and abdomen distension. He did not have a fever, weight loss, hematemesis, melena, or altered bowel habits. The patient was not on any chronic medications and had no significant family history. He did not have any recent travel history, followed a regular healthy diet, and did not smoke or consume alcohol. On examination, he was afebrile, and his vital signs were within normal limits. On physical examination, he had abdomen distension with dullness at bilateral flanks and diminished air entry in the right lung lower lobe. The patient underwent extensive laboratory investigations (Table 1). Initial blood count showed leukocytosis with hypereisnophilia 51%, slight elevation in CRP, and Ig-E was high at 269 IU/mL (Table 1). CT of the abdomen revealed generalized circumferential edematous wall thickening of the small bowel loops and to a lesser extent caecum and ascending colon with mesenteric congestion, reactive lymph nodes, mild ascites, and right-sided pleural effusion (Figures 5-7).

The patient was admitted to the hospital and started on intravenous hydration, PPI, antiemetic, and ciprofloxacin with metronidazole empirically. The Helicobacter pylori (H. pylori) urea breath test was positive. His initial autoimmune workup was reactive (Table 2). Ascites fluid and pleural fluid analysis showed predominant eosinophilia (Table 3). The patient was reviewed by a rheumatologist and several repeated antinuclear antibody tests were negative. The patient had an EGD that showed esophagitis, gastritis, and duodenitis. A colonoscopy showed erythematous mucosa in the terminal ileum, otherwise entire examined colon was normal. Histopathology results were significant for eosinophilic infiltration of the stomach, duodenum, terminal ileum, rectum, and random samples from the colon (Table 4). There was no evidence of malignancy from the various biopsy samples. Based on the patient’s clinical presentation and histopathology findings, he was diagnosed with EGE.

**Table 3:** Ascites and pleural fluid analysis

	Body fluid analysis			
	Pleural fluid	Reference range	Ascitic fluid	Reference range
White blood cell	5,013 * 10^6^	<5 * 10^6^	2,610 * 10^6^	<300 * 10^6^
Polymorphs	95%	-	73.5%	-
Neutrophils	2.0%	-	1.0%	-
Lymphocytes	8.0%	-	1.0%	-
Monocytes	40.0%	-	8.0%	-
Eosinophils	50.0%	-	88.9%	-
Albumin	-	-	30 g/L	-
Lactate dehydrogenase	110 IU/L	-	-	-
Total protein	48 g/L	-	40 g/L	-
Body fluid culture	Negative	-	Negative	-
Pathology	Reactive inflammatory response, negative for malignancy	-	Reactive inflammatory response, negative for malignancy	-

**Table 4:** Eosinophilic count per high power field from several GI tract biopsies

Stomach	Mild acute gastritis with some features suggestive of reactive reflux/chemical gastropathy; only scattered eosinophils seen
Duodenum	Conspicuous eosinophilic infiltration (> 52 eosinophils/high power field); focal clusters of eosinophils in lamina propria and muscularis mucosae
Terminal ileum	Conspicuous eosinophilic infiltration (> 56 eosinophils/high power field) with focal mild acute inflammation
Colon	Conspicuous eosinophilic inflammation (> 60 eosinophils/high power field) with patchy mild acute inflammation and occasional neutrophilic and eosinophilic cryptitis; rare neutrophilic crypt abscesses noted
Rectum	Patchy mild eosinophilic infiltrate (< 10 eosinophils/high power field)

The patient remained well during hospitalization with good response following PPI and intravenous hydration. He had mild improvement in the symptoms and peripheral eosinophilia. He was started on steroids, six-elimination diets, and H. pylori eradication therapy with outpatient clinic follow-up. Following two months of tapering steroids, he was initiated on mepolizumab. The patient had his follow-up in December 2023 after three injections of Mepolizumab 100mg q4 weeks. The patient is in complete remission with a resolution of his GI symptoms. Moreover, a recent EGD and colonoscopy showed marked improvement in the inflammatory process involving the upper GI tract and no histological evidence of eosinophilic colitis. Also, he had magnetic resonance enterorrhaphy of the small bowel that reported normal study with complete resolution of ascites, pleural effusion, and previous diffuse small bowel thickening. In February 2024, a trial of stopping mepolizumab failed, and the patient developed severe reflux symptoms and food intolerance. He was re-initiated on biologics and as of his last follow-up in July 2024, the patient is symptom-free with a good response to mepolizumab.

## Discussion

EGE is an IgE-mediated disorder characterized by eosinophil-rich inflammation of the GI tract [5]. The pathogenesis of EGE is unclear, but numerous studies indicated an overproduction of cytokines-secreting T helper type 2 effector cells, chemokines, eotaxin-1, interleukin-5 (IL-5), and interleukin-15, which leads to the upregulation of eosinophils and infiltration of the GI tract causing a cascade of inflammatory response [1,3,5]. According to Klein’s classification, the clinical presentation and symptoms vary based on the affected site and layer of the GI tract infiltrated by eosinophils [2,6]. Mucosal layer involvement is the most common and patients can develop symptoms such as abdominal pain, vomiting, or diarrhea [1,5]. Other patients can present with symptoms of intestinal obstruction if the muscular layer is affected in the disease process [1,5]. The rarest is the sub-serosal layer infiltration causing peritonitis, ascites, and pleural effusion in patients with EGE [1,5,6]. The majority of EGE cases have peripheral eosinophilia, elevated IgE levels, and raised erythrocyte sedimentation rate, and if having serositis, the fluid will be exudative with eosinophilic predominance [1]. Both of our patients had mild CRP elevation and negative ESR even during periods of relapse. Patients can present with an array of clinical presentations, but the definitive diagnosis relies on histological examination of the involved GI tract, revealing evidence of eosinophilic infiltration [1]. The second patient in our case report had both upper GI endoscopy and colonoscopy, with eosinophilic infiltration found throughout the GI tract. It is important that patients with EGE are screened thoroughly with multiple biopsy sites.

Various therapeutic options have been proposed and demonstrated to be effective, including PPI, H. pylori eradication, dietary intervention, corticosteroids, mast cell stabilizers, leukotriene receptor antagonists, immunomodulators, surgery, and biologic agents [2,4]. Corticosteroids continue to be the mainstay of treatment for EGE and have proven efficacious in all age groups, however, no randomized controlled trials (RCTs) exist for treatment efficacy comparison. The first line for induction is typically prednisolone at a dose of 30-40 mg/day, followed by a one to three-month gradual taper [7]. The prolonged use of steroids has various adverse effects and biologics act as a steroid-sparing agent to achieve remission in patients with EGE. Different biological agents have been mentioned in the literature for the treatment of eosinophilic GI diseases such as mepolizumab, cendakimab, dupilumab, and omalizumab [8]. In our cases, we utilized the use of mepolizumab, an anti-IL5- antibody, at a dose of 100mg every four weeks. Our patients have shown significant and rapid improvement with complete remission from the first few doses of mepolizumab. The complete remission was monitored both histologically and clinically. IL-5 cytokine regulates the growth and lifecycle of eosinophils and is incorporated into the pathogenesis of EGE [9]. Mepolizumab attaches itself to IL-5 and prevents IL-5 signaling, which lowers eosinophil production and survival [9]. The reduction in blood eosinophilia will therefore control the clinical symptoms in patients diagnosed with EGE. Mepolizumab is FDA-approved as an add-on therapy for treating severe eosinophilic asthma, eosinophilic granulomatosis with polyangiitis (EGPA), and hyper-eosinophilic syndrome [6,9]. Not enough data is available regarding its effect on EGE; however, two RCTs showed improvement in eosinophilic count in patients with eosinophilic esophagitis despite limited improvement in symptoms [4]. A case report mentioned in the literature showed that mepolizumab can lead to remission within five months of initiating treatment in patients with EGE [6]. Another case report concluded that mepolizumab can be effective in patients with EGE associated with asthma [10] and this can correlate with our first case which is currently in remission for more than two years on mepolizumab.

The natural history and prognosis of EGE are still not yet fully understood and require further research. EGE typically exhibits a wax-and-wane pattern, and usually, flares can be managed effectively [6]. A higher risk of relapse is reported in patients with high eosinophilic count, especially with mucosal involvement of the proximal small intestine [6]. One potential strategy to stop EGE recurrences is to implement an elimination diet [11]. Around 40% of the cases require surgery at some point in the course of their disease due to surgical complications like perforation and intestinal obstruction [2]. Overall, available data suggest that EGE has a favorable prognosis, with a low risk of malignancy [4,6]. Our second patient tested positive for autoantibodies but did not exhibit any specific autoimmune disease symptoms. Autoantibody positivity has been associated with EGE, and autoimmune illness may manifest later in life, according to published research [11].

## Conclusions

The diagnosis of EGE can be challenging due to the vast array of symptoms that patients may experience. Additionally, limited data in the Middle East region poses a challenge in approaching EGE patients. No definite diagnostic criteria have been established; therefore, physicians need to be more vigilant with patients at high risk of developing the disease. The advancement in diagnosis and management has provided several options in the management of EGE. Biologic agents, with limited adverse effects, can be safely used in EGE. However, the limitation is the availability and cost of the medication. Currently, no specific indicators exist to predict disease progression; hence, patients should be carefully monitored for GI symptom relapses, and interval endoscopy can be beneficial in determining treatment response. Our cases will help provide more insight into the diagnosis and management of EGE, particularly in the UAE.
